# State-of-the-Art Deep Learning Methods for Objects Detection in Remote Sensing Satellite Images

**DOI:** 10.3390/s23135849

**Published:** 2023-06-23

**Authors:** Adekanmi Adeyinka Adegun, Jean Vincent Fonou Dombeu, Serestina Viriri, John Odindi

**Affiliations:** 1 School of Mathematics, Statistics and Computer Science, University of KwaZulu-Natal, Durban 4041, South Africa; adeguna@ukzn.ac.za; 2 School of Mathematics, Statistics and Computer Science, University of KwaZulu-Natal, Pietermaritzburg 3209, South Africa; fonoudombeuj@ukzn.ac.za; 3 School of Agricultural, Earth and Environmental Sciences, University of KwaZulu-Natal, Pietermaritzburg 3209, South Africa; odindij@ukzn.ac.za

**Keywords:** remote sensing, satellite images, object detection, YOLO, R-CNN

## Abstract

**Introduction:** Object detection in remotely sensed satellite images is critical to socio-economic, bio-physical, and environmental monitoring, necessary for the prevention of natural disasters such as flooding and fires, socio-economic service delivery, and general urban and rural planning and management. Whereas deep learning approaches have recently gained popularity in remotely sensed image analysis, they have been unable to efficiently detect image objects due to complex landscape heterogeneity, high inter-class similarity and intra-class diversity, and difficulty in acquiring suitable training data that represents the complexities, among others. **Methods:** To address these challenges, this study employed multi-object detection deep learning algorithms with a transfer learning approach on remotely sensed satellite imagery captured on a heterogeneous landscape. In the study, a new dataset of diverse features with five object classes collected from Google Earth Engine in various locations in southern KwaZulu-Natal province in South Africa was used to evaluate the models. The dataset images were characterized with objects that have varying sizes and resolutions. Five (5) object detection methods based on R-CNN and YOLO architectures were investigated via experiments on our newly created dataset. **Conclusions:** This paper provides a comprehensive performance evaluation and analysis of the recent deep learning-based object detection methods for detecting objects in high-resolution remote sensing satellite images. The models were also evaluated on two publicly available datasets: Visdron and PASCAL VOC2007. Results showed that the highest detection accuracy of the vegetation and swimming pool instances was more than 90%, and the fastest detection speed 0.2 ms was observed in YOLOv8.

## 1. Introduction

Remotely sensed satellite image analysis is critical for a range of applications that include land cover mapping, urban planning, disaster management, and environmental monitoring [1]. Specifically, the identification of objects such as residential buildings and water bodies from remotely sensed satellite images is crucial for, among other things, landscape planning and management and preventing and mitigating disasters such as flooding and fires [2]. However, object detection from remotely sensed satellite images has remained a challenge due to complexities that include large image size, varying lighting conditions, and complex backgrounds [3,4]. While deep learning methods have recently shown remarkable performance in object detection and identification tasks, these methods have been inefficient for image analysis due to insufficient training datasets that depict landscape complexity [5]. In recent years, researchers have proposed different Convolutional Neural Networks (CNN)-based architectures such as Faster Region CNN (R-CNN), You Only Look Once (YOLO), and Retina Net for object detection in satellite images [6,7]. These architectures employ different techniques, such as region proposal, anchor boxes, and feature pyramid networks to optimize object detection in images [7]. R-CNN is a popular Region-based Convolutional Neural Network (R-CNN), which first proposes object regions and then executes a classification [7,8]. Despite deep learning methods showing great potential for the identification and detection of image objects [9], their adoption has been impeded by the often complex image features and limited training datasets. Hence, further research is needed to address the challenges of object detection and recognition in imagery, and to develop more robust and accurate deep learning models for relevant applications. Commonly, several challenges characterize the use of deep learning methods for image object detection and recognition. Such challenges include high variability in object appearance, the presence of noise and artifacts, and the limited availability of annotated data [9,10].

These challenges, which include the complex characteristics of remotely sensed images and the limited availability of labeled datasets for training deep learning-based object detection methods, are discussed below.

### 1.1. Complex Features of Remote Sensing Images

Object detection is a challenging task that involves identifying and localizing objects within an image. However, there are several limitations to existing object detection methods that include complex object characteristics, image backgrounds, and instance annotation. There are also challenges relating to high intra-class and low inter-class variance [11,12,13].

- Varying sizes, structures and resolutions: Objects in remotely sensed imagery are characterized by varying sizes and resolutions, high inter-class similarity, and intra-class diversity [14]. This presents a challenge for object detection methods, as they must accurately identify objects of different sizes and shapes within the image. Additionally, the high inter-class similarity and intra-class diversity make it difficult to distinguish between different object classes, further complicating the object detection process. Furthermore, a high degree of similarity may occur among objects in images that are intensely similar [11], making extracting similar features confusing to detectors, hence incorrect outcomes. Recent studies have focused on developing object detection methods specifically for satellite images. These include the use of multi-scale object detection techniques and deep learning-based methods that can handle complex object characteristics [14]. In this study, we established the effects of training models on a novel dataset created from environmental perception data characterized by diverse features and captured in multiple scenes and on the performance of such models.
- Challenging background: The background of an image can present challenges for object detection methods, as it causes difficulty in distinguishing between objects and their surroundings. This is particularly true in remotely sensed satellite imagery, where the background can be highly variable and contain similar texture and color patterns to the objects of interest. Various methods have been adopted to handle complex image backgrounds. These include the use of contextual information such as incorporating contextual features into the object detection process and the use of object proposal methods to pre-select potential object regions within the image. However, these methods are yet to achieve optimum performance [15,16].
- Limited labeled dataset: Instance annotation refers to the process of labeling individual objects within an image with their respective class labels and bounding boxes. However, this process can be complex and time-consuming, particularly in cases where there are large numbers of objects within the image or when the objects have complex shapes or occlusions [17]. This can lead to errors in the annotation process, which can negatively impact the accuracy of the object detection method. Inaccurate sample annotations were therefore established in this study as a major factor that increases the complexity of detection implementation. Applications such as urban monitoring, disaster prediction, and general environmental monitoring require an accurate and effective object detection approach.

In this paper, to address these challenges, we proposed a newly created dataset of diverse images characterized by objects of varying sizes and resolutions acquired under real-life conditions and quality, and with high inter-class similarity and intra-class diversity. The dataset was further subjected to data augmentation, to increase the diversity of training data. This study employed a transfer learning and dynamic data fusion approach for modeling state-of-the-art deep learning-based object detection methods on the newly created dataset for improved performance. Five popular object detection algorithms (Detectron2, YOLOv5, YOLOv6, YOLOv7, and YOLOv8) based on R-CNN and YOLO were modeled and evaluated on the proposed dataset. The transfer learning approach involves using pre-trained models on large-scale datasets to improve the performance of object detection and recognition in remote sensing satellite images. The study was an experimental of state-of-the-art review on image object detection.

### 1.2. Our Approach

In this study, the approaches are summarized as follows.

- Design of a novel dataset from environmental perception data characterized with diverse features and captured in multiple scenes;
- Review of related works;
- Modeling R-CNN and YOLO-based algorithms on the newly created dataset;
- Conducting experiments to establish the object detection performance of the state-of-the-art object detection algorithms.

## 2. Review of Related Works

The accurate detection of objects in remotely sensed images is critical in socio-economic and biophysical mapping and monitoring [18]. This process involves identifying and detecting specific locations of objects of interest in satellite images. In recent years, state-of-the-art machine learning and deep learning techniques have been used to effectively detect and recognize small objects in satellite imagery [19]. Some of the recently deployed techniques include Faster-RCNN, Single Shot Detector (SSD), and YOLO [20]. Faster RCNN is a deep learning-based approach that relies on the Region Proposal Network (RPN) algorithm, the SSD framework relies on the extraction of feature maps through different layers that are later utilized for object detection using CNN filters, and YOLO employs the CNN algorithm for object detection [20,21]. Wang et al. [22] proposed a method for the detection of buildings from imagery using a combination of a CNN and a long short term memory (LSTM) network. CNN was used to extract features from the images and LSTM was used to model the spatial relationships between features. Additionally, a deep learning-based method was proposed to detect ships from synthetic aperture radar (SAR) images [23]. Using CNN to extract features from the images and a region proposal network (RPN), the results showed that the proposed method outperformed several existing deep learning systems. A region proposal-based method [24], Mask R-CNN, was used to detect aeroplanes and ships in images. The system used a mask branch to describe objects’ shapes and a Feature Pyramid Network (FPN) module to improve the detection of small objects. Yang Long et al. [25] employed region-based techniques for object localization from images, while Van et al. [26] deployed Satellite Imagery Multiscale Rapid Detection with Windowed Networks (SIMRDWN), an updated version of You Only Look Twice (YOLT), along with faster RCNN and SSD for object detection. The model was evaluated on satellite imagery and achieved a 0.2 km^2^/s rate at detecting vehicle objects. Adam et al. [27] proposed YOLT, a two-stage object detection method based on YOLO that employs both coarse- and fine-grained detection stages to generate regions of interest (ROIs) and to refine the ROIs for the classification of objects. Ku et al. [28] proposed a modified version of YOLOv4, YOLOv4-ISR, for capturing an image spatial relationship (ISR) module to improve object detection performance in images. The model was deployed for real-time object detection in a factory. Sun et al. [29] proposed a version of YOLOv4, Auto-T-YOLO, for detecting objects in images. The model was evaluated on the publicly available SAR ship detection dataset (SSDD).

Some advanced object detection systems have also been developed in the recent past. Lei et al. [30] introduced an algorithm that combines CNN and a transformer to tackle the issue of low detection rate and high false alarm rate in ship detection using Synthetic Aperture Radar (SAR) images corrupted by noise. Xu et al. [31] integrated Swin-Transformer into one-stage frameworks to achieve real-time ship target detection in maritime environments. They evaluated their system on a sea-ships dataset and obtained an average precision score of 80.59%. Their approach also served as the backbone for YOLOv3 and SSD frameworks. Zhang et al. [32] proposed a Ship Detection Transformer called ESDT, which utilizes ResNet50 as the backbone for feature extraction. They incorporated encoder multi-scale self-attention to capture long-range dependencies in the features and employed a decoder for final ship detection. The system was adapted to learn from the large pretrained DETR model and tested on the commonly-used ship detection dataset, Seaships. Chen et al. [33] presented a ship detection model, CSD-YOLO, based on YOLOv7 and designed for complex scenes. They introduced an SAS-FPN module that combines atrous spatial pyramid pooling and shuffle attention to enhance detection accuracy and the model’s capability to detect objects at various scales. The model’s performance was evaluated on the HRSID and SSDD datasets. An Automatic Ship Detection (ASD) approach utilizing deep learning (DL) methods was developed to analyze the Airbus ship dataset. Different YOLO algorithms, including YOLOv3, YOLOv4, and YOLOv5, were experimented with using a large satellite image dataset from the Airbus Ship Challenge and Shipsnet [34]. A lightweight variant called YOLOV5-MNE was created by modifying the MNEBlock module using standard CBR convolutions and incorporating the CA (coordinate attention) mechanism to enhance detection performance [35]. The model achieved a precision of 94.7% on the SAR ship detection (SSDD) dataset. To improve ship detection in SAR images, Nambiar et al. [36] proposed an approach that integrates advanced deep learning techniques with a deepSORT tracking algorithm. They explored and evaluated various models, including Faster-RCNN, YOLOv5, G-CNN, and SSD, on publicly available SAR datasets. They conducted experiments on a newly created custom dataset called the Lateral Ship Detection Dataset (LSDD). Lastly, a YOLO-based system was deployed [37] for detecting objects in images. The system employs a differential model with channel attention layers for finding the anchor configurations and outperformed some existing algorithms by 3.58% and 5.13% on public datasets, DIOR and RSOD. Some state-of-the-art object detection methods and their limitations are summarized in Table 1:

## 3. Methods

### 3.1. Methods Overview

**Figure 1:**
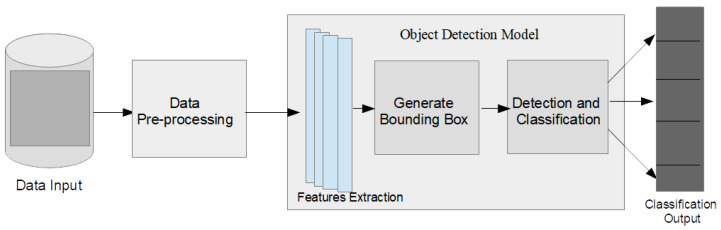
Architectural diagram for the proposed methodology.

This section presents an overview of the proposed methodology (Figure 1). The figure describes the process pipeline: dataset creation and pre-processing, model architectures, and training and learning processes for the models. The final results predicted are sent into the classification output. The architectures of the models used in this study are further elaborated in this section. In the study, five deep learning methods based on R-CNN and YOLO have been experimented with. The processes involved in our methodology are highlighted and discussed below:

- Dataset creation and preprocessing;
- Model architectures;
- Model training—transfer learning.

### 3.2. Dataset Creation

The dataset creation process is the first step of the object detection pipeline as presented in Figure 1. This process is critical because the performance and accuracy of deep learning models are dependent on the quality and quantity of the dataset used. Effective object detection in images requires a vast and quality dataset. Recent advances in technology have motivated the creation of customized large-scale datasets for developing deep learning models for the detection of objects in specific locations from images.

**Figure 2:**
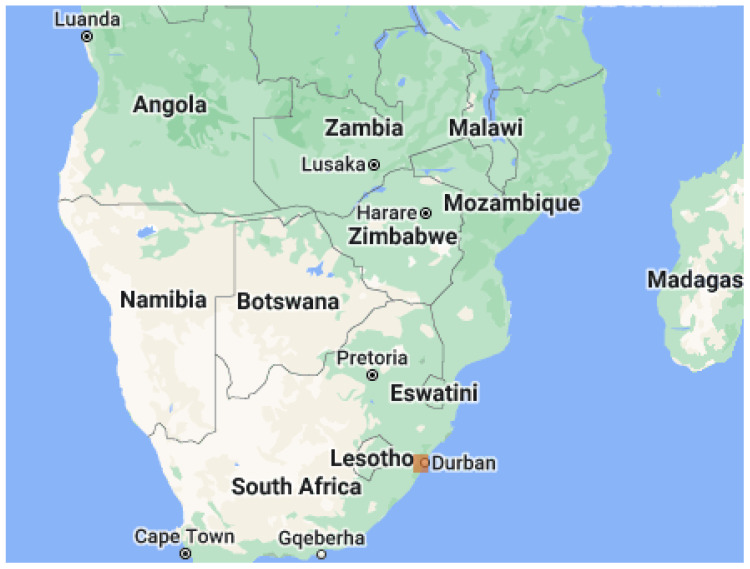
A diagram showing extracted region for dataset acquisition from larger map view [40].

**Figure 3:**
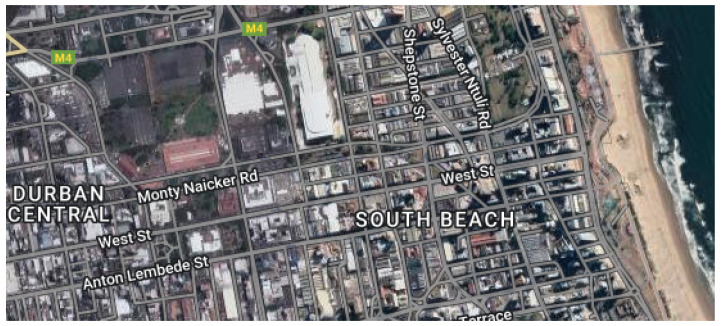
Region of Interest (ROI) identification for dataset collection [40].

In this study, we used the Google Earth Engine [39,40] to acquire images and set them at a resolution of 640 × 640 pixels. The Engine was used to acquire Sentinel-2 MSI imagery collected from the southern part of Durban city in the Kwazulu-Natal province of South Africa (coordinates: latitude −29.8579 and longitude 31.0292). The images were captured from the imagery at 640 × 640 dimensions each. Roboflow [41], an end-to-end computer vision platform, was employed to organize and annotate the images and create datasets. The dataset collection regions are presented in Figure 2 and Figure 3.

**Figure 4:**
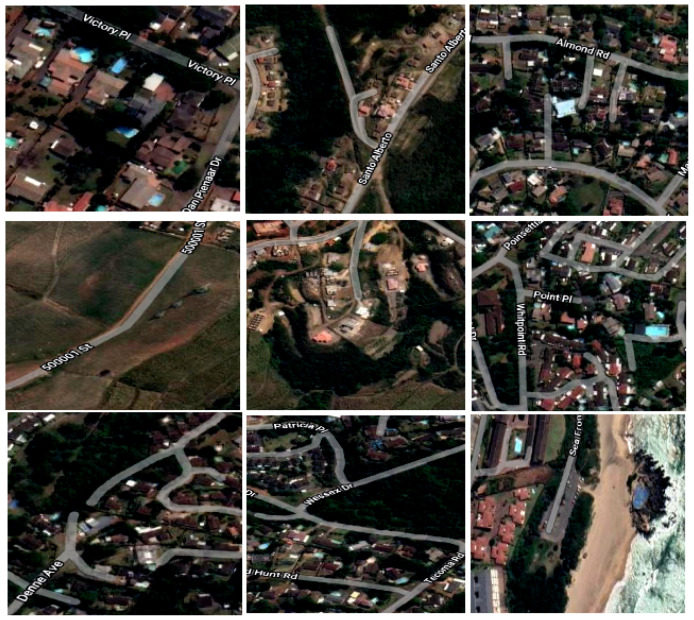
Example of images from Training dataset.

The dataset includes 92 satellite images, which were annotated in the multi-class classification format. The images contain five objects; residence, roads, shoreline, swimming pool, and vegetation. Sample images from the dataset are presented in Figure 4. The dataset was further categorized into three datasets: training, validating, and testing, with the training set containing 61 images and one annotation file, the validation set containing 21 images and one annotation file, and the testing set containing 10 images. Some pre-processing tasks were applied to the images including auto-orientation of pixel data. The training dataset was then subjected to image augmentation to increase the quantity.

We employed a data augmentation approach to the proposed dataset to enhance the performance of the models in the detection accuracy and robustness. The approach employs geometric augmentations and transformations that change the spatial orientation of images but do not change the content. This helps to diversify the training set and make the models more resilient to changes in perspective or orientation. It involves flipping the image horizontally to create a mirror image and flipping it vertically to invert the image. It also involves rotating the images by 90, 180, or 270 degrees to simulate the different viewing angles of an object in the image. These processes were performed repeatedly to increase the quantity of the training images by 100 times.

**Figure 5:**
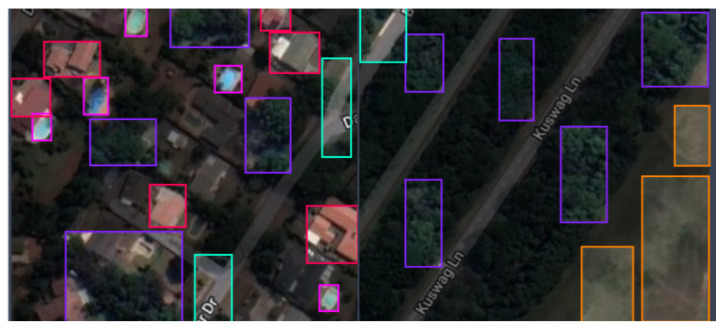
Labeling images for objects detection.

The objects are evenly distributed in the images in the final datasets with each of the objects identified and annotated four to five times on each image in the final dataset. An example of how different objects were annotated is represented in Figure 5.

### 3.3. Generalized Objects Detection Systems Architecture

**Figure 6:**
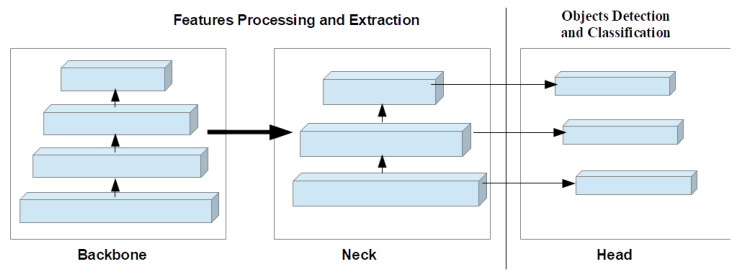
An illustration of backbone, neck and head composition in Generalized Object Detection Systems Architecture.

Most deep learning-based object detection methods are made up of three main components—the head (prediction section), neck (feature pyramid network), and backbone (features extraction network) as presented in Figure 6 and described below:

- Head: The head module is the final component of the architecture and is responsible for predicting the bounding boxes, class probabilities, and objectness scores for each object in the input image [42]. The head module takes the feature maps generated by the neck module and applies a set of convolutional filters to predict the locations and sizes of bounding boxes for each object. The head is composed of several fully connected layers that perform regression and classification tasks. The head section, for example, predicts the location and size of objects using anchor boxes and applies a softmax activation function to output class probabilities. The head module can also predict the class probabilities for each bounding box, indicating the object class corresponding to the bounding box. The head module predicts the objectness score for each bounding box, indicating the likelihood that the bounding box contains an object [43,44,45]. The output of the head module is always a set of bounding boxes, each with a corresponding class and objectness score.
- Backbone: The backbone is the core network architecture that processes the input image and extracts high-level features that are useful for detecting objects [46]. Typically, the backbone consists of several layers of CNNs, such as convolutional and pooling layers and activation functions, which are stacked on top of each other. These layers perform operations such as convolution, pooling, and activation to gradually reduce the spatial resolution of the input image while increasing its depth. The YOLO architecture [47], for example, uses popular backbones such as Darknet-53 or ResNet as the backbone. The output of the backbone is a set of feature maps that capture different levels of abstraction and spatial scale.
- Neck: The neck module connects the feature pyramids to the head of the architecture [48]. Feature pyramids are multi-scale feature maps that are generated by the backbone network [49]. These feature maps contain information about objects at different scales and resolutions, allowing the architecture to detect objects of different sizes. They are created by applying a set of convolutional filters and spatial pooling to the output of the backbone at different scales. The feature pyramids are used to detect objects of different sizes and scales. The neck region is composed of a series of convolutional and pooling layers, which are used to upsample an extracted features map from the backbone region. A common example of the neck region is the feature pyramids network [50].

### 3.4. Network Architectures

The model series employed in the research, which include R-CNN-based Detectron2, YOLOv5, YOLOv6, YOLOv7, and YOLOv8 are presented in Table 2. Detectron2 architecture is generally based on R-CNN frameworks and pretrained on datasets, which include Common Object in Context (COCO) and Pascal VOC [51]. The Detectron2 framework is made up of the following components: data loading, backbone network, feature pyramid network (FPN), region proposal network (RPN), and detection head. The YOLO series are generally composed of three main sections: backbone, neck and head. The backbone is responsible for extracting features from the input image, the neck combines features from different levels of the backbone, and the head generates object detection predictions.

YOLOv5 also uses a “Swish” activation function, which is faster and more accurate than the traditional “ReLU” function used in previous YOLO versions. YOLOv6 explores both regression and classification losses: VariFocal loss and an SIoU, GIoU. It also explores a quantization scheme using RepOptimizer and channel-wise distillation in the head section. YOLOv5 and YOLOv6 employ the Cross Stage Partial Networks (CSP) modules with other modules at the backbone and neck regions. YOLOv7 employs Concatenation based Models and Efficient Layer Aggregation Network at the backbone section. It also uses Path Aggregation Network (PAN)-based FPN in the neck region and employs multiple heads: lead and auxiliary heads. YOLOv5 and YOLOv8 use anchor-free object detection, which eliminates the need for anchor boxes, and a dynamic anchor assignment method.

YOLOv8 architecture presents a framework with a reduction in the number of parameters and the overall size of the tensors by replacing the CSP modules in the neck and backbone regions with a novel C2f module. This has led to faster computation, reduced computational resources, and efficient detection in YOLOv8 as presented in our results.

### 3.5. Model Network Training—Transfer Learning

In this study, we adopted a transfer learning approach [59] to improve the effectiveness of our models. Specifically, this involved pre-training the models on a larger dataset, which contains a vast collection of images of common objects in daily life. By pre-training our models on a large and diverse dataset, we could leverage the wealth of information available to create a more generalized feature extractor. After pre-training, we then retrained the models on our newly created dataset. The classes in the proposed dataset were the primary target tasks for customization in this study. By retraining the models on these specific classes, we fine-tuned the models to improve their accuracy and performance for the target tasks. Overall, this approach enabled us to leverage the vast amount of data available to create a strong feature extractor. This, in turn, allowed fine-tuning our models on the newly created dataset more efficiently to achieve better accuracy and generalization.

## 4. Results and Discussion

### 4.1. Evaluation Metrics

The deep learning-based object detection methods were evaluated on our custom dataset. The analysis of the models was based on five metrics—detection accuracy (DA), precision (P), average precision (AP), mean average precision (mAP), and recall (R). These are presented below:

- Detection accuracy is a measure of how well a model can correctly identify objects within an image. There are several metrics used to evaluate the detection accuracy of a model; these include precision, recall, and F1-score;
- Precision refers to the proportion of true positives (correctly detected objects) out of all the objects detected by the model. A high precision indicates that the model has a low rate of false positives, meaning that most of the objects it detects are correct;
- Average precision (AP) is the average precision of the objects detected by the model;
- Mean average precision (mAP) is the overall mean value of the AP;
- Recall measures the proportion of true positives detected by the model out of all the actual objects in the image. A high recall indicates that the model can detect most of the objects in the image.

### 4.2. Results Discussion

#### 4.2.1. Experimental Analysis of State-of-the-Art

The proposed dataset was used to evaluate object detection performance using precision, recall, mAP50, and mAP (50:95) metrics. Table 3 presents the results, which show that the state-of-the-art detection methods perform competitively across all evaluation metrics. Specifically, YOLOv8 achieved 68%, 60%, 43%, and 17.5% in precision, recall, mAP50, and mAP (50:95), respectively, while maintaining the highest speed limit of 0.2 ms. Although YOLOv5, YOLOv6, and YOLOv7 showed marginal improvement, they performed similarly. Detectron2 achieved a precision score of 50% but at a slower rate than the YOLO-based models.

#### 4.2.2. Class-Wise Detection Performance

Table 4 illustrates the class-wise detection performance of YOLOv8 on the proposed dataset, identifying the five objects—Residence, Roads, Shorelines, Swimming Pool, and Vegetation—from the test image samples. Among these objects, Swimming Pool demonstrated the highest precision score of 62.7%, followed by Vegetation with a precision score of 57.3% and Shorelines with a precision score of 54.6%. Swimming Pool also achieved the highest recall score of 62.9%, followed by Shorelines with a recall score of 62.4% and Vegetation with a recall score of 62.3%. Residence and Roads exhibited similar detection rates, with Residence achieving scores of 41.1%, 42.1%, 19.3%, and 12.8% for precision, recall, mAP50, and mAP (50:95), respectively, and Roads achieving scores of 41.2%, 57.1%, 13.7%, and 4.75% for the same metrics. This similarity in detection rates is attributed to their similar visual appearance. The model demonstrated a better detection performance for Shorelines, Swimming Pool, and Vegetation, which can be attributed to their larger sizes and resolutions as well as the quality of the background.

#### 4.2.3. Visualization Analysis of Detection Results Output

**Figure 7:**
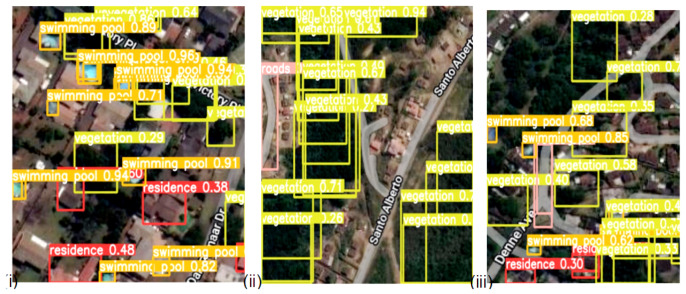
Detecting dense objects from sample satellite image testing datasets; (**i**) Detection of Swimming Pools, Vegetation, and Residences; (**ii**) Detection of dense objects such as Vegetation; (**iii**) Detection of Vegetation and Swimming Pools.

**Figure 8:**
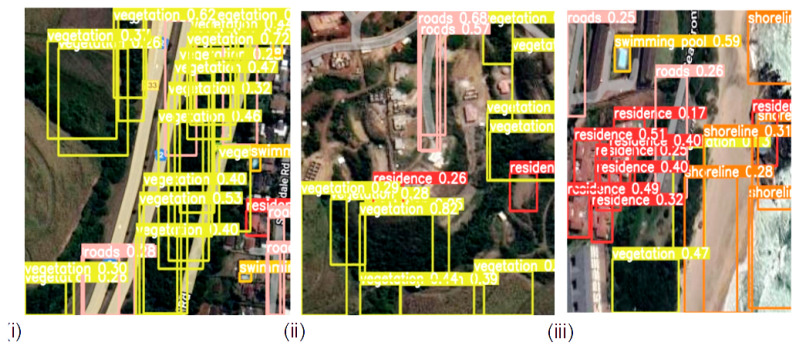
Densely Distributed objects detection from sample satellite image testing datasets; (**i**) Detecting clustered vegetation objects; (**ii**) Detecting clustered vegetation and roads objects; (**iii**) Detecting clustered residence, shorelines and roads objects.

**Figure 9:**
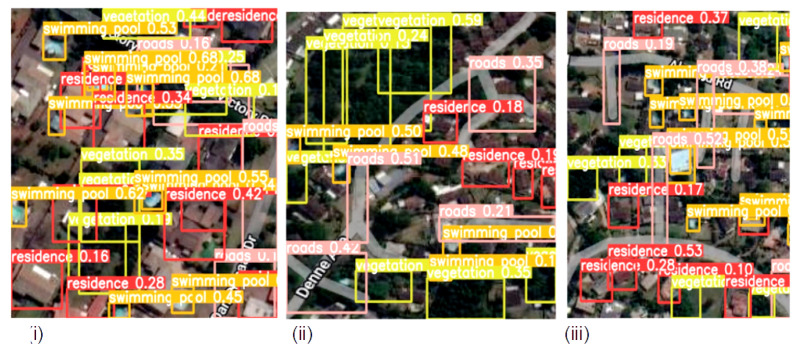
Evenly distributed objects detection from sample satellite testing datasets; (**i**) Detecting swimming pools, residence and vegetation objects; (**ii**) Detecting vegetation, swimming pools and residence objects; (**iii**) Detecting residence, swimming pools and roads objects.

**Figure 10:**
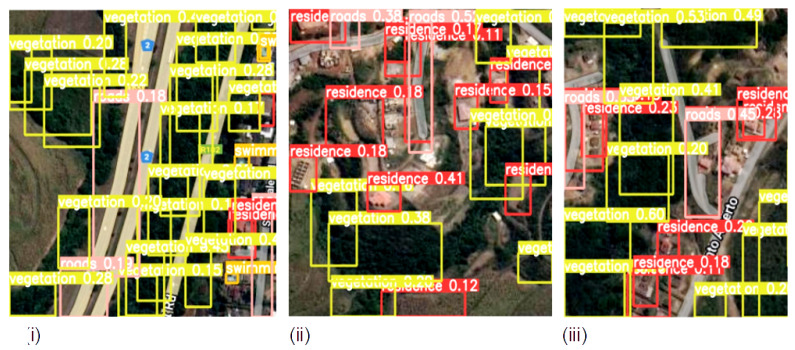
Detecting evenly and densely distributed objects from sample satellite image testing datasets; (**i**) Detecting densely distributed vegetation and evenly distributed residence objects; (**ii**) Detecting evenly distributed residence and vegetation objects; (**iii**) Detecting evenly distributed residence, vegetation and roads objects.

**Figure 11:**
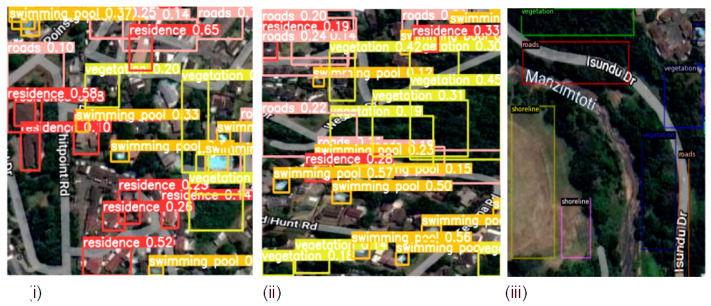
More Evenly Distributed objects detection from sample satellite images (**i**) Detecting evenly distributed residence, roads, vegetation and swimming pools objects; (**ii**) Detecting evenly distributed roads, residence and vegetation objects; (**iii**) Detecting slightly and densely distributed shorelines, vegetation and roads objects.

**Figure 12:**
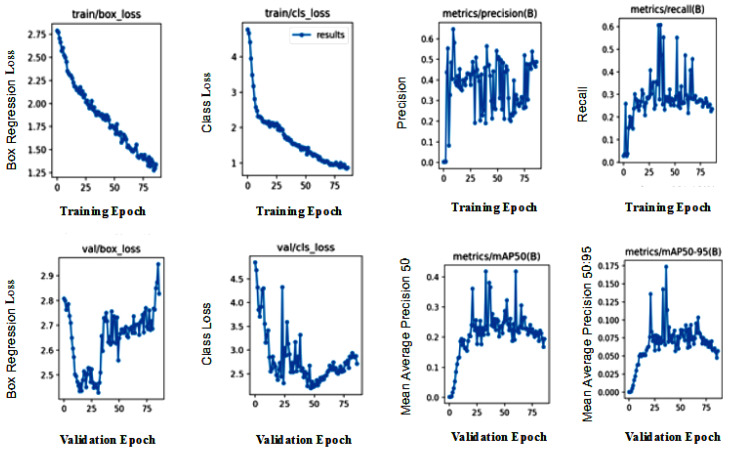
Performance evaluation curves of YOLOv8 on the proposed dataset; First row presents training curves for box regression loss, class loss, precision, and recall; Second row presents validation curves for box regression loss, class loss, mean average precision:50, and mean average precision: 50:95 dataset.

The detection results output are represented by the bounding boxes and detected objects. Figure 7i presents a detection accuracy of 59% for Swimming Pools, 47% for Vegetation, and 51% for Residences. Shorelines and Roads were also well-detected. Figure 8i,iii confirm the detection of more Swimming Pools, whereas Figure 9ii depicts the better detection of Roads. Furthermore, the model demonstrated better detection of dense objects, such as Vegetation, in Figure 7ii,iii and Figure 8i,ii, Residences in Figure 9i,iii, and Shorelines in Figure 8iii. From the result outputs analysis, it can be established that Vegetation was detected the most, followed by Swimming Pools and Shorelines. This implies that dense objects are easily detected. The results also reveal that Vegetation achieved the highest detection accuracy of 97% and Swimming Pools achieved a detection accuracy of 96%. The examples of detection results in Figure 10i–iii and Figure 11 show that objects can be precisely detected even when they are evenly distributed and dense as can be seen in the examples of Vegetation and Residence. The detection algorithms perform well through Figure 7, Figure 8, Figure 9, Figure 10 and Figure 11 with almost all objects successfully detected even in the presence of high density overlap between neighboring objects. Lastly, the overall YOLOv8 performance on the proposed dataset is also illustrated with precision, average precision, recall, and error plots for the models in Figure 12. From the figure, the model achieved more than 60% in both precision and recall. It also achieved more than 50% in mAP. The loss curves show steady training for the model and that the model performed well on training data. This is a positive sign that the model trained effectively on the proposed dataset.

#### 4.2.4. Comparison Analysis of the Models on Publicly Available Datasets: Visdrones and Pascalvoc

According to a comparison analysis of different models tested on two datasets, Pascal VOC2007 [60] and VisDrones [61], YOLOv8 displayed a superior performance with the highest mAP value. The details of these datasets are outlined in Table 5, while Table 6 and Table 7 present the results obtained from testing YOLOv3, YOLOv5, YOLOv7, and YOLOv8 on the respective datasets. The outcomes demonstrate that YOLOv8 outperforms the other models, even for small and normal-sized target objects.

### 4.3. Summary and Future Works

#### 4.3.1. Summary

Three major challenges have been highlighted in this research. The approaches adopted in addressing the challenges are summarized below:

- Varying sizes, structures, and resolutions:
  – In this paper, to address the challenges of varying sizes, structures, and resolutions, we proposed a newly-created dataset of diverse images characterized by objects of varying sizes and resolutions acquired under real-life conditions and quality, and with high inter-class similarity and intra-class diversity. We established the effects of training models on a novel dataset created from environmental perception data characterized by diverse features and captured in multiple scenes on the performance of the models.
  – The data augmentation approach employed during training enabled the model to efficiently handle objects with varying sizes, structures, and resolutions.
  – Multi-scale training: The proposed system also employed a multi-scale training strategy that enables the model to learn representations at different scales. This enhances the ability to handle objects of varying sizes, structures, and resolutions.
- Challenging background: The research addresses the issue of challenging backgrounds through the following approaches:
  – Data augmentation: Data augmentation approaches employed expose the model to a wider range of backgrounds and help the object detecting model to generalize better and improve its ability to handle challenging backgrounds during inference.
  – The object detecting system adopted in this paper has numerous layers and components that learn to recognize patterns and objects of varying complexity and challenging background. For example, the backbone has a series of convolutional layers that extract relevant features from the input image, SPPF layer, and the subsequent convolution layers that process features at a variety of scales. The C2f module combines the high-level features extracted with contextual information to improve detection accuracy. The C2f component in the head structure is succeeded by two decoupled segmentation heads, which learn to predict the semantic segmentation masks for the input image. The Detection module uses a set of convolution and linear layers to map the high-dimensional features to the output bounding boxes and object classes.
- Limited labeled dataset:
  – The dataset was subjected to data augmentation, to increase the diversity of training data. The approach employs geometric augmentations and transformations that change the spatial orientation of images. This helps to diversify the training set and make the models more resilient to changes in perspective or orientation. It involves flipping the image horizontally to create a mirror image and flipping it vertically to invert the image. It also involves rotating images by 90, 180, or 270 degrees to simulate different viewing angles of an object in the image. These processes were performed repeatedly to increase the quantity of the training images by 100 times.
  – This study also employed a transfer learning and dynamic data fusion approach for modeling the object detection method on the newly created dataset for improved performance. The transfer learning approach involves using pre-trained models on large-scale datasets to improve the performance of object detection and recognition in remote sensing satellite images.

#### 4.3.2. Future Works

Even though this research has been able to resolve the challenges identified, some objects were undetected. The proposed object detection framework still struggles with detecting very small objects, in particular the ones clustered together with some occlusions. Improving localization accuracy still remains challenging. Further enhancement to improve localization and detection accuracy for very small objects with the presence of challenging occlusions will be explored in future research. Recent advancements in deep learning such as an ensemble deep learning approach, integrating vision transformer and attention-based mechanisms to enhance the model ability in detecting objects with complex characteristics will be explored to improve the localization accuracy. This will generally improve detection accuracy and average precision score.

## 5. Conclusions

In this paper, an experimental study on the performance of state-of-the-art techniques for detecting objects in remote sensing satellite images has been carried out. The research identified some complex features in remote sensing images, such as high inter-class similarity, intra-class diversity, and challenging background, as limitations of the state-of-the-art in detecting objects in remote sensing satellite images. The research proposed a newly-created dataset and provides a comprehensive performance evaluation and analysis of recent deep learning-based methods for object detection in high-resolution remote sensing satellite images via experimental research with a transfer learning approach. The performance of these methods is promising with YOLOv8, achieving 68% and 60% in precision and recall when evaluated on the proposed dataset. A comparison analysis of object detection methods on Pascal VOC and Visdrone datasets was also performed, with a superior performance from YOLOv8.

## Figures and Tables

**Table 1:** Performance of object detection methods on various datasets and their limitations.

Methods	Datasets	Results Obtained	Limitations
FasterR-CNN [38]	VOC2007	mAP 76.4%, 5 frames per seconds (fps)	Difficulty in detecting small objects and real-time detection
SPP-Net [38]	VOC2007	54.2% mAP	Requires complicated multi-step training steps and large computational resources, lower detection rate and accuracy
R-CNN [38]	VOC2007	66%, 0.5 fps.	calculation efficiency is too low, may cause object distortion, lower detection rate
FasterR-CNN [20]	AGs-GF1 and 2	86.0%, 12 fps	Lower detection accuracy
YOLOv3 [20]	AGs-GF1 and 2	90.4%, 73 fps	Slower detection rate
SSD [20]	AGs-GF1 and 2	84.9%, 35 fps	Lower detection accuracy
YOLOv4 [19]	MS COCO	43.5%, 65.7 fps	Requires larger computational resources
YOLOv5s [21]	SIMD	5.8 ms, 62.8 mAP	Lower detection speed and accuracy
YOLO-HR [21]	SIMD	67.31% mAP, 6.7 ms	Requires larger computational resources, Lower detection speed and accuracy
RetinaNet [23]	SAR	average precision 79%	Difficulty in detecting smaller objects
YOLOv3 [23]	SAR	63% mAP	Requires larger computational resources, lower detection speed and accuracy
YOLT [26]	DigitalGlobe satellites, planet satellites, and aerial platforms.	68% mAP, 44 fps	Difficulty in detecting small objects, lower detection speed and accuracy
YOLOv4-Tiny [37]	RSOD	80.02% mAP, 285.3 fps	Lower detection speed s
YOLOv4 + CSPA + DE [37]	RSOD	85.13% mAP, 227.9 fps	Challenges of obtaining biased anchors due to the large variation in object scales in remote sensing images.

**Table 2:** Description of Models Architectures.

Methods	Backbone Structure	Neck Structure	Head Structure
Detectron2	The backbone network is responsible for extracting features from the input image. Detectron2 provides various backbone architectures such as ResNet, ResNeXt, and MobileNet.	Neck has FPN, a top-down architecture using multi-scale features, taking backbone output, and creating feature maps at different scales. It also contains RPN, generating object regions in an input image from feature maps.	The detection head takes the features extracted from ROI pooling and produces the final detection results. It consists of two sub-components: classification and regression.
YOLOv5	YOLOv5 [52,53] utilizes a Cross Stage Partial hybridized with Dark-net53 (CSP-Darknet53) as the backbone. The structure aims at limiting the vanishing gradient challenge with a dense system.	The neck uses Spatial Pyramid Pooling (SPP) and Path Aggregation Network with BottleNeckCSP (PANet) [54] for enhanced information flow through information aggregation.	The head is made up of a series of convolution layers for the prediction of bounding boxes and object classes score.
YOLOv6	It employs a backbone based on RepVGG called EfficientRep that uses a higher parallelism than previous YOLO backbones [55].	The neck uses PAN enhanced with RepBlocks or CSPStackRep Blocks for the larger models [55].	The head adopts new loss modules (VariFocal, SIoU, GIoU) and uses RepOptimizer-based quantization and channel-wise distillation for better detection [55].
YOLOv7	The backbone employs concatenation-based models (CBS) and Efficient Layer Aggregation Network (E-ELAN) algorithms [55] for feature extraction [56,57] and efficient learning and convergence.	The neck structure employs a PAN-based feature pyramid network. This allows the system to efficiently manage the transmission of both high-level and low-level features and enhance the accuracy.	YOLOv7 incorporates Deep Supervision, a technique that employs multiple heads, including the lead head responsible for the final output, and an auxiliary head that assists with training in middle layers.
YOLOv8	The backbone [55,58] includes convolution layers, coarse-to-fine (C2f) modules, and spatial pyramid pooling faster (SPPF) modules. Bottleneck components extend to neck regions and feature maps; $C_{n}$ and $P_{n}$ are extracted from the backbone and neck regions.	Neck region concatenates features for fewer parameters and tensor size. YOLOv8 uses the C2f module in the neck region, replacing CSP and C3 modules with “f” denoting feature count.	The C2f component in the head structure is succeeded by two decoupled segmentation heads. The detection heads in YOLOv8 are composed of detection modules and a prediction layer and are also anchor-free detection.

**Table 3:** Models performance on the proposed dataset.

Methods	P (%)	R (%)	mAP50 (%)	MAP50-95 (%)	Speed (ms)
Detectron2	50	32.7	16	24	0.9
YOLOv5	53.4	49.7	27	18.4	0.5
YOLOv6	53.2	47.4	32.1	16.6	0.4
YOLOv7	54.5	46.2	34.1	25	0.3
YOLOv8	68	60	43	17.5	0.2

**Table 4:** Class-wise performance of YOLOv8 on the proposed dataset.

Class	P (%)	R (%)	mAP50 (%)	MAP50-95 (%)
Residence	41.1	42.1	19.3	12.8
Roads	41.2	57.1	13.7	4.75
Shorelines	54.6	96.4	99.5	59.7
Swimming Pool	62.7	62.9	45.5	12.8
Vegetation	57.3	62.3	12.8	8.45

**Table 5:** Visdrone and PascalVOC datasets description.

Datasets	No of Images	Categories	Description
PascalVOC2007 [13]	9963	20	Person: person Animal: sheep, bird, horse, cat, dog, cow Vehicle: aeroplane, train, bicycle, motorbike, boat, car, bus Indoor: bottle, sofa, chair, potted plant, dining table, tv/monitor
VisDrones [60]	10,209	10	pedestrian, tricycle, truck, van, car, person, motorcycle, bus, awning, and bicycle

**Table 6:** Comparison analysis of object detection methods on Visdrone dataset [61].

Methods	mAP50 (%)
YOLOv3	38.8
YOLOv5	38.1
YOLOv7	30.7
YOLOv8	39.0

**Table 7:** Comparison analysis of object detection methods on Pascal VOC dataset [61].

Methods	mAP50 (%)
YOLOv3	79.5
YOLOv5	78
YOLOv7	69.1
YOLOv8	83.1

## Data Availability

Bench-marked data sets used is available in public repository.
